# Facteurs de risque de mortalité par tuberculose pulmonaire

**DOI:** 10.11604/pamj.2014.19.347.5321

**Published:** 2014-12-03

**Authors:** Hicham Janah, Hicham Souhi, Hatim Kouismi, Karima Mark, Rachida Zahraoui, Jouda Benamor, Mona Soualhi, Jamal Eddine Bourkadi

**Affiliations:** 1 Service de Phtisiologie de l'Hôpital Moulay Youssef, CHU Rabat, Maroc

**Keywords:** Tuberculose, Mortalité, Comorbidité, Tuberculosis, Mortality, Comorbidity

## Abstract

La tuberculose est une maladie infectieuse transmissible provoquée par myco-bacterium tuberculosis (bacille de Koch ou BK). Elle représente, selon les estimations del'Organisation Mondiale de la Santé (OMS), l'une des pathologies infectieuses causant le plus de décès au niveau mondial avec plus de 1 million de décès par an. Pour déterminer les facteurs de risque de mortalité au cours de la tuberculose pulmonaire à microscopie positive nous avons mené une étude rétrospective portant sur tous les cas de tuberculose pulmonaire à microscopie positive et qui étaient décédés au cours de leur hospitalisation. Cette étude a colligé 1803 cas de tuberculose sur une période de 2 ans et demi dont 46 sont décédés. La prévalence de décès est de 2,55%. La population se répartit en 32 hommes et 14 femmes. L’âge moyen était de 53ans ± 17 ans. Le tabagisme était retrouvé chez la moitié des cas. Une comorbidité était retrouvée dans 43%, avec 17% de diabète. Le délai de diagnostic avait une médiane de 60 jours avec percentile (30j; 105j). La symptomatologie clinique était dominée par la toux, la dyspnée et les expectorations soit respectivement: 97,8%, 69,6% et 67,4% des cas. Sur le plan radiologique les lésions étaient diffuses et bilatérales dans 76,1% des cas. Tous les patients étaient mis sous SRHZ. 11% avaient présenté une toxicité aux antibacillaires (de type hépatiques dans 3 cas et neurologiques dans 2 cas). Le délai médian de décès était de 8,5 jours (5j; 17j). Les causes de décès retrouvées étaient: Une hépatite fulminante (3 cas), une décompensation acido-cétosique (3 cas), un SDRA (2 cas), des hémoptysies foudroyantes (2 cas), et respectivement un cas secondaire à une décompensation de BPCO, une décompensation cardiaque, une hypoglycémie et un tableau d'anasarque. Cette étude suggère que le terrain, le retard diagnostique et les effets secondaires du traitement sont les principaux facteurs de risque de mortalité chez les patients hospitalisés pour tuberculose pulmonaire.

## Introduction

La tuberculose demeure un problème de santé publique pour une grande partie de la population mondiale. Il s'agit de la deuxième cause de décès par maladies infectieuses après l'infection par le virus d'immunodéficience humaine (VIH). L'Organisation Mondiale de la santé (OMS), recense en 2012, 8,6 millions de nouveaux cas de la maladie avec 1,3millions de décès. Près de 85% des nouveaux cas de TB ont été diagnostiqués en Afrique sub-saharienne et en Asie du Sud [1]. Le but de ce travail est de déterminer les facteurs de risque de mortalité au cours de la tuberculose pulmonaire à microscopie positive.

## Méthodes

Il s'agit d'une étude rétrospective portant sur tous les cas de tuberculose pulmonaire à microscopie positive et qui étaient décédés au cours de leur hospitalisation. Cette étude a colligé 1803 cas de tuberculose dont 46 décès sur une période de 2 ans et demi.

**Critère d'inclusion:** tous les nouveaux cas de tuberculose pulmonaire à microscopie positive sous traitement antituberculeux qui étaient décédés au cours de leur hospitalisation.

**Critères d'exclusion:** étaient exclus de l’étude les cas de rechute de tuberculose pulmonaire, d’échec ou de résistance et les patients ayant un antécédent de traitement antituberculeux.

Nous avons ainsi étudié les données épidémiologiques, cliniques, des examens complémentaires (biologiques, radiologiques, bactériologiques), des moyens thérapeutiques, des modalités évolutives. L’étude statistique était réalisée grâce au logiciel SPSS version 18. Les effectifs sont exprimés en pourcentage. Les valeurs quantitatives sont exprimées en moyenne et écart type quand la distribution est symétrique et par la médiane et quartile.

## Résultats

Au cours de la période de sélection, parmi 1803 patients tuberculeux hospitalisés, on a relevé 46 décès soit 2,55%. Il s'agissait de 32 hommes et de 14 femmes, (Sex-ratio = 2,28). L’âge moyen de 53 ans±17 ans. La moitié des cas étaient tabagiques. 43% des cas avaient au moins une pathologie associée, Le diabète était la comorbidité la plus retrouvée (17%) suivie par la BPCO dans 15% des cas. Des comorbidités cardiovasculaires (HTA, insuffisance cardiaque, trouble du rythme cardiaque. . .) étaient retrouvées chez 7% des cas. La sérologie HIV était positive chez 2% des cas. Le délai de diagnostic avait une médiane de 60 jours avec quartile de (30j; 105j). La symptomatologie clinique était dominée par la toux, la dyspnée et les expectorations soit respectivement: 97,8%, 69,6% et 67,4%. Les signes généraux étaient présents chez 97,8% (Tableau 1).

**Tableau 1:** Répartition des patients selon les caractéristiques cliniques

Signes cliniques	Nombre de patients (%)
**Signes respiratoires**	
Toux	97,8
Dyspnée	69,6
Expectorations	67,4
Hémoptysie	23,4
Douleurs thoraciques	9,6
**Signes généraux**	
Altération de l’état général	96,3
Fièvre	84,6
Sueurs	73,4

**Figure 1:**
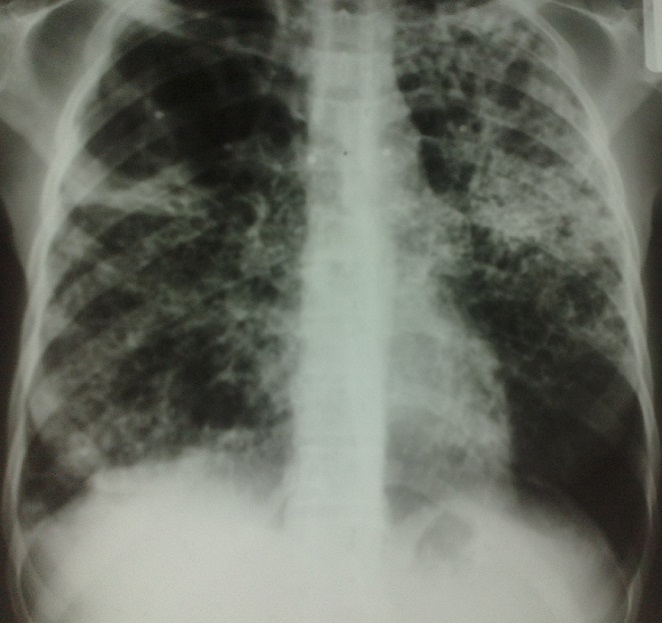
Radiographie du thorax, lésions pulmonaires excavées étendues et bilatérales chez un BPCO

**Figure 2:**
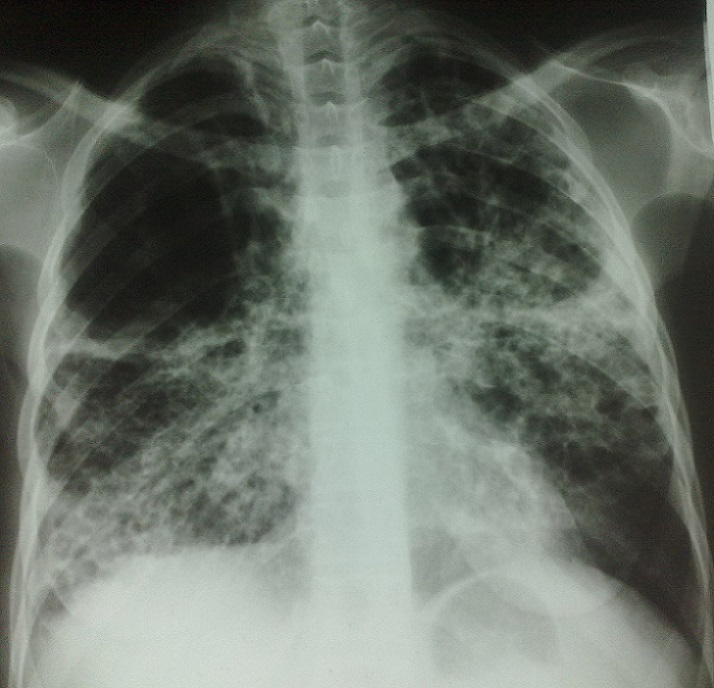
Radiographie du thorax, Tuberculose pulmonaire étendue et bilatérale chez une femme

Sur le plan radiologique les opacités excavées sont retrouvées chez 35% des cas, les infiltrats réticulonodulaires chez 46% et l'association des deux aspects chez 67% des cas. Les lésions étaient diffuses et bilatérales chez 76% des cas (Figure 1 et Figure 2).

Tous les patients avaient reçu un traitement antituberculeux quadruple à base de (isoniazide, rifampicine, pyrazina¬mide et streptomycine). 11% des cas avaient présenté une toxicité aux antibacillaires: hépatique dans 3 cas et neurologique dans 2 cas.

Le délai médian de décès était de 8,5 jours avec quartile de (5j; 17j). Les causes de décès retrouvées étaient: Une hépatite fulminante (3 cas), une décompensation acido-cétosique (3 cas), un SDRA (2 cas), des hémoptysies foudroyantes (2 cas), et respectivement un cas secondaire à une décompensation de BPCO, une décompensation cardiaque, une hypoglycémie et un tableau d'anasarque.

## Discussion

La tuberculose est une maladie infectieuse ancienne, qui redevient d'actualité, le nombre de nouveaux cas déclarés ayant régulièrement augmenté aux tours des dernières années. Ce phénomène de réémergence, incluant le rôle favorisant du VIH, et la dégradation des conditions socioéconomiques dans certains pays s'associent à l'apparition de souches multirésistantes aux antituberculeux classiques.

La tuberculose représente la huitième cause de décès dans les pays à revenu faible et intermédiaire (la septième chez l'homme et la neuvième chez la femme). Chez les adultes âgés de15 à 59 ans, elle représente la troisième cause de décès, après le VIH/sida et la cardiopathie ischémique. En 2012, 1,3 millions de personnes sont décédées de la TB; près d'un million étaient séronégatives pour le VIH et 320 000 séropositives. La région africaine compte le nombre de décès le plus important [1, 2]. Dans notre série, l’âge moyen retrouvé est inférieur à celui décrit aux pays industrialisé, ceci peut être expliqué par le fait que la tuberculose au Maroc touche les sujets jeunes. Avec une surmortalité masculine.

Mais globalement, nos données rejoignent celle de l'incidence actuelle de la maladie dans les pays a forte prévalence, notamment en Afrique ou l'on observe, dune part, une plus grande incidence masculine et, d'autre part. Une incidence maximale pour les catégories d’âge avancé. L'incidence minimale pour les grands enfants et adolescents est justifiée pour certains par la dure de la couverture vaccinale par le BCG qui ne protègerait de la maladie qu'une quinzaine d'années, expliquant la reprise de l'infection chez les adultes jeunes [3]. Les facteurs qui ont été reconnus comme responsables de l'actuelle surmortalité masculine sont des facteurs à la fois génétiques et environnementaux [4]. Le tabagisme dans la moitié des cas, ce facteur de risque a été déjà cité par une équipe internationale qui avait démontré que le tabagisme est la cause majeure de la moitié des décès par tuberculose en Inde [5, 6].

Dans une revue de la littérature portant sur les déterminants du délai diagnostic au cours de la tuberculose, ce délai était relativement homogène à travers les différentes études sélectionnées, quelque soit le pays, situé entre 60 et 90 jours [7]. De manière attendue, les délais les plus longs étaient retrouvés dans les pays en voie de développement, à l'exception d'une étude effectuée dans la banlieue est de Londres où le délai diagnostic médian était de 126 jours [8].

Dans notre travail, le délai total était de 60 jours, proche des moyennes retrouvées dans la littérature. Il était comparable à des pays comme la Tunisie, Vietnam, l'Afrique du sud et le Nigéria [9–11]. Ce délai était plus long que celui retrouvé dans certaines études menées dans des pays à haut revenu tel que le Japon et la Chine (Hong Kong) [12, 13] Les lésions étaient diffuses chez la majorité des patients, en rapport avec le délai de diagnostic relativement long et la comorbidité. A noter que HIV n'est retrouvé que chez 2 malades alors que l'OMS dit que un malade sur 4 atteint d'HIV meurt par la tuberculose et Globalement, 320 000 personnes sont décédées par une coïnfection tuberculose/VIH en 2012 [1, 14]. Selon Citron [15] le déclin survenu de mortalité par tuberculose en Europe au début du XXème siècle serait à mettre en relation, d'une part, avec une meilleure immunité des populations et, d'autre part, avec une diminution du risque de diffusion de la maladie liée à l'amélioration des conditions socio-économiques. L'association entre mortalité par tuberculose et paupérisme a été soulignée a plusieurs reprises [16, 17]. La diminution de la mortalité par tuberculose est contemporaine de la réalisation de travaux d'urbanisme, de l'amélioration progressive des conditions de travail, de l'assainissement des logements et de l’élévation relative du niveau de vie.

## Conclusion

Au terme de ce travail, les comorbidités, le retard diagnostique et les effets secondaires du traitement sont les principaux facteurs de risque de mortalité chez les patients hospitalisés pour tuberculose pulmonaire. Au Maroc, le Programme National de Lutte Contre la Tuberculose instaure la gratuité absolue du dépistage, du diagnostic et du traitement de toutes les formes de TB dans les structures de santé publique, mais faut-il encore que le patient consulte et que la tuberculose soit évoquée par le médecin.
